# Diphenyl (*p*-tolyl­amido)­phosphate

**DOI:** 10.1107/S1600536810049354

**Published:** 2010-11-30

**Authors:** Mehrdad Pourayoubi, Poorya Zargaran, Shahriar Ghammamy, Hossein Eshtiagh-Hosseini

**Affiliations:** a Department of Chemistry, Ferdowsi University of Mashhad, Mashhad 91779, Iran; b Department of Chemistry, Faculty of Science, Imam Khomeini International University, Qazvin, Iran

## Abstract

The P atom in the title compound, C_19_H_18_NO_3_P, exhibits a distorted tetra­hedral configuration while the N atom shows a planar coordination. In the crystal, inter­molecular N—H⋯O hydrogen bonds form centrosymmetric dimers.

## Related literature

The reaction of compounds having phospho­rus-halide bonds with primary and secondary amines results in formation of phospho­rus-nitro­gen compounds, see: Chivers *et al.* (2003 ▶). For amido­phospho­ric acid esters (APEs), see: Gholivand *et al.* (2007 ▶); Ghadimi *et al.* (2007 ▶). For applications of APEs, see: Bao *et al.* (1993 ▶); Ghadimi *et al.* (2008 ▶); Nguyen & Kim (2008 ▶).
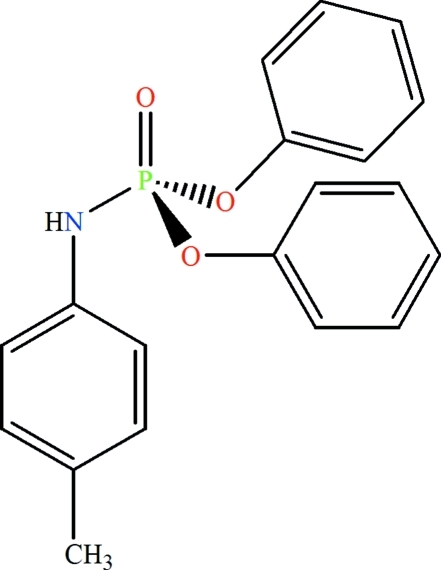


## Experimental

### 

#### Crystal data

C_19_H_18_NO_3_P
*M* *_r_* = 339.31
Triclinic, 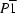

*a* = 9.7406 (10) Å
*b* = 9.9653 (10) Å
*c* = 11.1788 (12) Å
α = 96.337 (2)°
β = 109.303 (2)°
γ = 117.827 (2)°
*V* = 858.93 (15) Å^3^
*Z* = 2
Mo *K*α radiation
μ = 0.18 mm^−1^
*T* = 120 K
0.24 × 0.21 × 0.11 mm

#### Data collection

Bruker SMART 1000 CCD area-detector diffractometer
Absorption correction: multi-scan (*SADABS*; Sheldrick, 1996 ▶) *T*_min_ = 0.980, *T*_max_ = 0.989
8165 measured reflections
3744 independent reflections
3232 reflections with *I* > 2σ(*I*)
*R*_int_ = 0.020

#### Refinement

*R*[*F* ^2^ > 2σ(*F* ^2^)] = 0.045
*wR*(*F* ^2^) = 0.099
*S* = 1.01
3744 reflections
218 parameters
H-atom parameters constrained
Δρ_max_ = 0.31 e Å^−3^
Δρ_min_ = −0.57 e Å^−3^

### 

Data collection: *SMART* (Bruker, 1998 ▶); cell refinement: *SAINT-Plus* (Bruker, 1998 ▶); data reduction: *SAINT-Plus*; program(s) used to solve structure: *SHELXS97* (Sheldrick, 2008 ▶); program(s) used to refine structure: *SHELXL97* (Sheldrick, 2008 ▶); molecular graphics: *SHELXTL* (Sheldrick, 2008 ▶); software used to prepare material for publication: *SHELXTL*.

## Supplementary Material

Crystal structure: contains datablocks I, global. DOI: 10.1107/S1600536810049354/ng5073sup1.cif

Structure factors: contains datablocks I. DOI: 10.1107/S1600536810049354/ng5073Isup2.hkl

Additional supplementary materials:  crystallographic information; 3D view; checkCIF report

## Figures and Tables

**Table 1:** Hydrogen-bond geometry (Å, °)

*D*—H⋯*A*	*D*—H	H⋯*A*	*D*⋯*A*	*D*—H⋯*A*
N1—H1⋯O1^i^	0.79	2.07	2.840 (3)	167

Symmetry code: (i) 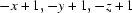.

**Fig. 1.**
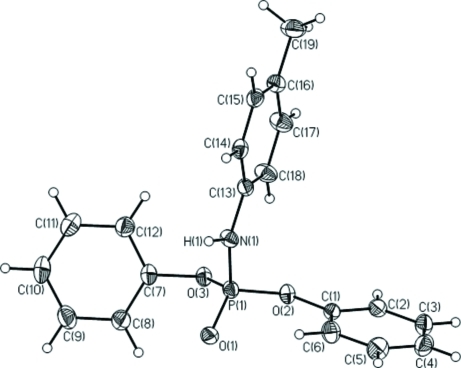
Molecular view with the atom labeling scheme, displacement ellipsoids are drawn at the 50% probability level. H atoms are represented as small spheres of arbitrary radii.

**Fig. 2.**
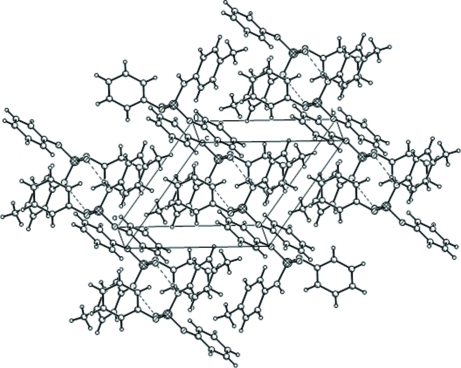
Partial packing view showing the formation of dimer through N–H···O interaction. H bonds are shown as dashed lines.

## supplementary crystallographic information

### Comment

The reaction of compounds having phosphorus-halide bonds with primary and secondary amines results in formation of phosphorus-nitrogen compounds (Chivers *et al.*, 2003). Amidophosphoric acid esters (APEs), a family of these compounds, contain an (O)(O)P(═O)(N) or (O)P(═O)(N)(N) skeleton which may be obtained from some initial phosphorus substances such as (RO)P(O)Cl_2_ and (N*R*^1^*R*^2^)(RO)P(O)Cl (Gholivand *et al.*, 2007; Ghadimi *et al.*, 2007). APEs have attracted attention because of the flame retardancy for their bisphosphorus derivatives (Nguyen & Kim, 2008), chiral catalyst preparation (Bao *et al.*, 1993) and biological properties (Ghadimi *et al.*, 2008).

Here, we report on the synthesis and single-crystal X-ray determination of title APE compound (Fig. 1); single crystals were obtained from CHCl_3_/n-C_7_H_16_ at r. t.

The phosphorus atom has a distorted tetrahedral configuration with the bond angles in the range of 94.65 (7)° [O(2)–P(1)–O(3)] to 117.14 (8)° [O(1)–P(1)–O(2)]. In crystal lattice, the H-bonded centrosymmetric dimer is formed *via* an intermolecular PO···HN hydrogen bond (N···O = 2.840 (3) Å). A fragment of unit cell packing showing the hydrogen bond is presented in Fig. 2.

### Experimental

To a solution of (C_6_H_5_O)_2_P(O)Cl in chloroform, a solution of *p*-toluidine and triethylamine (1:1:1 mole ratio) in chloroform was added at 273 K. After 4 h stirring, the solvent was removed and product was washed with distilled water and recrystallized from chloroform/n-heptane at room temperature. IR (KBr, cm^-1^): 3170.6 (NH), 3057.7, 2939.8, 2866.6, 2713.5, 2618.1, 2385.3, 1952.3, 1884.4, 1786.4, 1729.0, 1597.2, 1490.3, 1393.0, 1279.7, 1229.7, 1175.6, 1069.5, 974.8, 891.6, 819.2, 762.4, 681.6. Raman (cm^-1^): 3066.5, 3018.3, 2933.4, 1616.1, 1589.1, 1382.7, 1288.2, 1253.5, 1216.9, 1191.8, 1170.6, 1068.4, 1027.9, 1006.6, 941.1, 904.5, 837.0, 781.0, 759.8, 730.9, 636.4, 615.2, 590.1, 368.3, 333.6. ^31^P{^1^H} NMR (202.45 MHz, DMSO-d6, 300.0 K, H_3_PO_4_ external): -6.36 p.p.m. (*s*). ^1^H NMR (500.13 MHz, DMSO-d6, 300.0 K, TMS): 2.21 (*s*, 3H, CH_3_), 7.09 (*s*, 4H, Ar—H), 7.19–7.23 (*m*, 6H, Ar—H), 7.37–7.40 (*m*, 4H, Ar—H), 8.70 p.p.m. (*d*, ^2^J(P,H) = 10.6 Hz, 1H, NH). ^13^C NMR (125.75 MHz, DMSO-d6, 300.0 K, TMS): 20.16 (*s*, 1 C, CH_3_), 117.86 (*d*, ^3^J(P,*C*) = 7.7 Hz, 2 C, C*_ortho_*), 120.05 (*d*, ^3^J(P,*C*) = 4.8 Hz, 4 C, C*_ortho_*), 125.14 (*s*), 129.60 (*s*), 129.87 (*s*), 130.44 (*s*), 137.09 (*s*), 150.11 p.p.m. (*d*, ^2^J(P,*C*) = 6.3 Hz, 2 C, C*_ipso_*).

### Refinement

The hydrogen atom of NH group was found in difference Fourier synthesis. The H(C) atom positions were calculated. The H(N) atom was refined in isotropic approximation in riding model, the H(C) atoms were refined in isotropic approximation in riding model with the *U*_iso_(H) parameters equal to 1.2 *U*_eq_(Xi) or 1.5 *U*_eq_(Cii), where U(Xi) are the equivalent thermal parameters of the NH and CH atoms and U(Cii) are the ones of the CH_3_ carbon atoms to which the corresponding H atoms are bonded.

### Crystal data

**Table:**

C_19_H_18_NO_3_P	*Z* = 2
*M**_r_* = 339.31	*F*(000) = 356
Triclinic, *P*1	*D*_x_ = 1.312 Mg m^−^^3^
*a* = 9.7406 (10) Å	Mo *K*α radiation, λ = 0.71073 Å
*b* = 9.9653 (10) Å	Cell parameters from 948 reflections
*c* = 11.1788 (12) Å	θ = 3–29°
α = 96.337 (2)°	µ = 0.18 mm^−^^1^
β = 109.303 (2)°	*T* = 120 K
γ = 117.827 (2)°	Prism, colorless
*V* = 858.93 (15) Å^3^	0.24 × 0.21 × 0.11 mm

### Data collection

**Table:**

Bruker SMART 1000 CCD area-detector diffractometer	3744 independent reflections
Radiation source: fine-focus sealed tube	3232 reflections with *I* > 2σ(*I*)
graphite	*R*_int_ = 0.020
phi and ω scans	θ_max_ = 27.0°, θ_min_ = 2.0°
Absorption correction: multi-scan (*SADABS*; Sheldrick, 1996)	*h* = −12→12
*T*_min_ = 0.980, *T*_max_ = 0.989	*k* = −12→12
8165 measured reflections	*l* = −14→14

### Refinement

**Table:**

Refinement on *F*^2^	Primary atom site location: structure-invariant direct methods
Least-squares matrix: full	Secondary atom site location: difference Fourier map
*R*[*F*^2^ > 2σ(*F*^2^)] = 0.045	Hydrogen site location: mixed
*wR*(*F*^2^) = 0.099	H-atom parameters constrained
*S* = 1.01	*w* = 1/[σ^2^(*F*_o_^2^) + (0.010*P*)^2^ + 1.4*P*] where *P* = (*F*_o_^2^ + 2*F*_c_^2^)/3
3744 reflections	(Δ/σ)_max_ < 0.001
218 parameters	Δρ_max_ = 0.31 e Å^−^^3^
0 restraints	Δρ_min_ = −0.56 e Å^−^^3^

### Special details

**Table:**

Geometry. All e.s.d.'s (except the e.s.d. in the dihedral angle between two l.s. planes) are estimated using the full covariance matrix. The cell e.s.d.'s are taken into account individually in the estimation of e.s.d.'s in distances, angles and torsion angles; correlations between e.s.d.'s in cell parameters are only used when they are defined by crystal symmetry. An approximate (isotropic) treatment of cell e.s.d.'s is used for estimating e.s.d.'s involving l.s. planes.
Refinement. Refinement of *F*^2^ against ALL reflections. The weighted *R*-factor *wR* and goodness of fit *S* are based on *F*^2^, conventional *R*-factors *R* are based on *F*, with *F* set to zero for negative *F*^2^. The threshold expression of *F*^2^ > σ(*F*^2^) is used only for calculating *R*-factors(gt) *etc*. and is not relevant to the choice of reflections for refinement. *R*-factors based on *F*^2^ are statistically about twice as large as those based on *F*, and *R*- factors based on ALL data will be even larger.

### Fractional atomic coordinates and isotropic or equivalent isotropic displacement parameters (Å^2^)

**Table:**

	*x*	*y*	*z*	*U*_iso_*/*U*_eq_	
P1	0.30226 (6)	0.32453 (5)	0.30029 (5)	0.01976 (12)	
O1	0.26650 (17)	0.38136 (15)	0.40642 (13)	0.0232 (3)	
O2	0.16664 (16)	0.14657 (15)	0.20459 (12)	0.0212 (3)	
O3	0.28250 (16)	0.40203 (15)	0.18341 (13)	0.0216 (3)	
N1	0.4912 (2)	0.34721 (19)	0.36132 (15)	0.0214 (3)	
H1	0.5535	0.4125	0.4324	0.026*	
C1	0.1005 (2)	0.0096 (2)	0.24317 (19)	0.0203 (4)	
C2	−0.0075 (2)	−0.1338 (2)	0.1388 (2)	0.0235 (4)	
H2A	−0.0313	−0.1345	0.0492	0.028*	
C3	−0.0801 (3)	−0.2761 (2)	0.1673 (2)	0.0296 (4)	
H3A	−0.1534	−0.3750	0.0968	0.036*	
C4	−0.0461 (3)	−0.2743 (3)	0.2985 (2)	0.0319 (5)	
H4A	−0.0961	−0.3719	0.3179	0.038*	
C5	0.0611 (3)	−0.1297 (3)	0.4011 (2)	0.0325 (5)	
H5A	0.0837	−0.1289	0.4907	0.039*	
C6	0.1362 (3)	0.0147 (2)	0.3750 (2)	0.0276 (4)	
H6A	0.2097	0.1137	0.4454	0.033*	
C7	0.3697 (2)	0.5708 (2)	0.22208 (19)	0.0227 (4)	
C8	0.2885 (3)	0.6432 (2)	0.2520 (2)	0.0300 (4)	
H8A	0.1772	0.5812	0.2487	0.036*	
C9	0.3736 (3)	0.8088 (3)	0.2869 (2)	0.0347 (5)	
H9A	0.3204	0.8612	0.3081	0.042*	
C10	0.5357 (3)	0.8980 (2)	0.2909 (2)	0.0335 (5)	
H10A	0.5929	1.0112	0.3143	0.040*	
C11	0.6148 (3)	0.8228 (3)	0.2610 (2)	0.0327 (5)	
H11A	0.7263	0.8845	0.2646	0.039*	
C12	0.5309 (3)	0.6567 (2)	0.2257 (2)	0.0272 (4)	
H12A	0.5838	0.6039	0.2046	0.033*	
C13	0.5718 (2)	0.3089 (2)	0.29081 (18)	0.0204 (4)	
C14	0.7373 (2)	0.3403 (2)	0.36440 (19)	0.0216 (4)	
H14A	0.7917	0.3854	0.4590	0.026*	
C15	0.8219 (2)	0.3056 (2)	0.2994 (2)	0.0238 (4)	
H15A	0.9342	0.3281	0.3504	0.029*	
C16	0.7450 (2)	0.2383 (2)	0.1608 (2)	0.0255 (4)	
C17	0.5803 (3)	0.2073 (3)	0.0896 (2)	0.0294 (4)	
H17A	0.5251	0.1608	−0.0049	0.035*	
C18	0.4949 (3)	0.2425 (3)	0.1528 (2)	0.0275 (4)	
H18A	0.3832	0.2212	0.1015	0.033*	
C19	0.8337 (3)	0.1978 (3)	0.0886 (2)	0.0363 (5)	
H19A	0.7571	0.1485	−0.0074	0.054*	
H19B	0.8614	0.1228	0.1227	0.054*	
H19C	0.9401	0.2957	0.1035	0.054*	

### Atomic displacement parameters (Å^2^)

**Table:**

	*U*^11^	*U*^22^	*U*^33^	*U*^12^	*U*^13^	*U*^23^
P1	0.0190 (2)	0.0172 (2)	0.0197 (2)	0.00924 (19)	0.00640 (18)	0.00282 (18)
O1	0.0218 (6)	0.0218 (7)	0.0239 (7)	0.0107 (6)	0.0102 (5)	0.0030 (5)
O2	0.0227 (6)	0.0166 (6)	0.0198 (6)	0.0095 (5)	0.0069 (5)	0.0035 (5)
O3	0.0215 (6)	0.0166 (6)	0.0225 (6)	0.0093 (5)	0.0070 (5)	0.0038 (5)
N1	0.0191 (7)	0.0228 (8)	0.0174 (7)	0.0118 (7)	0.0039 (6)	0.0000 (6)
C1	0.0181 (8)	0.0185 (8)	0.0253 (9)	0.0105 (7)	0.0094 (7)	0.0068 (7)
C2	0.0224 (9)	0.0226 (9)	0.0250 (9)	0.0123 (8)	0.0101 (8)	0.0051 (8)
C3	0.0259 (10)	0.0201 (9)	0.0355 (11)	0.0099 (8)	0.0108 (9)	0.0035 (8)
C4	0.0300 (11)	0.0246 (10)	0.0435 (12)	0.0140 (9)	0.0178 (10)	0.0162 (9)
C5	0.0370 (12)	0.0332 (11)	0.0314 (11)	0.0197 (10)	0.0166 (9)	0.0159 (9)
C6	0.0310 (10)	0.0241 (10)	0.0236 (10)	0.0140 (9)	0.0090 (8)	0.0057 (8)
C7	0.0240 (9)	0.0174 (9)	0.0229 (9)	0.0105 (8)	0.0071 (7)	0.0060 (7)
C8	0.0246 (10)	0.0244 (10)	0.0399 (12)	0.0135 (8)	0.0129 (9)	0.0090 (9)
C9	0.0365 (12)	0.0258 (10)	0.0448 (13)	0.0208 (10)	0.0152 (10)	0.0100 (9)
C10	0.0350 (11)	0.0187 (9)	0.0351 (11)	0.0108 (9)	0.0079 (9)	0.0092 (8)
C11	0.0270 (10)	0.0268 (10)	0.0369 (12)	0.0093 (9)	0.0128 (9)	0.0132 (9)
C12	0.0264 (10)	0.0252 (10)	0.0300 (10)	0.0136 (8)	0.0122 (8)	0.0098 (8)
C13	0.0198 (9)	0.0176 (8)	0.0229 (9)	0.0105 (7)	0.0082 (7)	0.0048 (7)
C14	0.0193 (9)	0.0165 (8)	0.0215 (9)	0.0077 (7)	0.0044 (7)	0.0038 (7)
C15	0.0166 (8)	0.0198 (9)	0.0300 (10)	0.0086 (7)	0.0068 (8)	0.0067 (8)
C16	0.0221 (9)	0.0235 (9)	0.0303 (10)	0.0126 (8)	0.0112 (8)	0.0054 (8)
C17	0.0286 (10)	0.0363 (11)	0.0223 (10)	0.0200 (9)	0.0082 (8)	0.0033 (8)
C18	0.0228 (9)	0.0351 (11)	0.0240 (10)	0.0188 (9)	0.0061 (8)	0.0037 (8)
C19	0.0263 (10)	0.0434 (13)	0.0379 (12)	0.0191 (10)	0.0148 (9)	0.0040 (10)

### Geometric parameters (Å, °)

**Table:**

P1—O1	1.4691 (14)	C8—H8A	0.9500
P1—O2	1.5793 (13)	C9—C10	1.385 (3)
P1—O3	1.5926 (14)	C9—H9A	0.9500
P1—N1	1.6279 (16)	C10—C11	1.386 (3)
O2—C1	1.399 (2)	C10—H10A	0.9500
O3—C7	1.414 (2)	C11—C12	1.394 (3)
N1—C13	1.417 (2)	C11—H11A	0.9500
N1—H1	0.7881	C12—H12A	0.9500
C1—C6	1.387 (3)	C13—C18	1.388 (3)
C1—C2	1.388 (3)	C13—C14	1.403 (3)
C2—C3	1.387 (3)	C14—C15	1.391 (3)
C2—H2A	0.9500	C14—H14A	0.9500
C3—C4	1.390 (3)	C15—C16	1.395 (3)
C3—H3A	0.9500	C15—H15A	0.9500
C4—C5	1.386 (3)	C16—C17	1.393 (3)
C4—H4A	0.9500	C16—C19	1.509 (3)
C5—C6	1.393 (3)	C17—C18	1.387 (3)
C5—H5A	0.9500	C17—H17A	0.9500
C6—H6A	0.9500	C18—H18A	0.9500
C7—C8	1.383 (3)	C19—H19A	0.9800
C7—C12	1.377 (3)	C19—H19B	0.9800
C8—C9	1.389 (3)	C19—H19C	0.9800
			
O1—P1—O2	117.14 (8)	C10—C9—H9A	119.8
O1—P1—O3	114.32 (8)	C8—C9—H9A	119.8
O2—P1—O3	94.65 (7)	C11—C10—C9	120.22 (19)
O1—P1—N1	111.29 (8)	C11—C10—H10A	119.9
O2—P1—N1	107.89 (8)	C9—C10—H10A	119.9
O3—P1—N1	110.35 (8)	C10—C11—C12	120.1 (2)
C1—O2—P1	126.66 (12)	C10—C11—H11A	119.9
C7—O3—P1	116.80 (11)	C12—C11—H11A	119.9
C13—N1—P1	127.92 (13)	C7—C12—C11	118.45 (19)
C13—N1—H1	115.8	C7—C12—H12A	120.8
P1—N1—H1	112.7	C11—C12—H12A	120.8
C6—C1—C2	121.82 (18)	C18—C13—C14	118.81 (17)
C6—C1—O2	123.22 (16)	C18—C13—N1	123.00 (16)
C2—C1—O2	114.95 (16)	C14—C13—N1	118.18 (16)
C3—C2—C1	119.12 (18)	C15—C14—C13	120.21 (17)
C3—C2—H2A	120.4	C15—C14—H14A	119.9
C1—C2—H2A	120.4	C13—C14—H14A	119.9
C4—C3—C2	120.23 (19)	C14—C15—C16	121.26 (17)
C4—C3—H3A	119.9	C14—C15—H15A	119.4
C2—C3—H3A	119.9	C16—C15—H15A	119.4
C3—C4—C5	119.69 (19)	C17—C16—C15	117.64 (18)
C3—C4—H4A	120.2	C17—C16—C19	120.30 (19)
C5—C4—H4A	120.2	C15—C16—C19	122.06 (18)
C6—C5—C4	121.1 (2)	C18—C17—C16	121.78 (19)
C6—C5—H5A	119.5	C18—C17—H17A	119.1
C4—C5—H5A	119.5	C16—C17—H17A	119.1
C1—C6—C5	118.05 (19)	C17—C18—C13	120.29 (18)
C1—C6—H6A	121.0	C17—C18—H18A	119.9
C5—C6—H6A	121.0	C13—C18—H18A	119.9
C8—C7—C12	122.44 (18)	C16—C19—H19A	109.5
C8—C7—O3	118.89 (17)	C16—C19—H19B	109.5
C12—C7—O3	118.65 (17)	H19A—C19—H19B	109.5
C7—C8—C9	118.42 (19)	C16—C19—H19C	109.5
C7—C8—H8A	120.8	H19A—C19—H19C	109.5
C9—C8—H8A	120.8	H19B—C19—H19C	109.5
C10—C9—C8	120.3 (2)		
			
O1—P1—O2—C1	−50.89 (16)	C12—C7—C8—C9	0.0 (3)
O3—P1—O2—C1	−171.29 (14)	O3—C7—C8—C9	−178.54 (18)
N1—P1—O2—C1	75.58 (15)	C7—C8—C9—C10	0.2 (3)
O1—P1—O3—C7	50.54 (15)	C8—C9—C10—C11	−0.4 (3)
O2—P1—O3—C7	173.16 (13)	C9—C10—C11—C12	0.5 (3)
N1—P1—O3—C7	−75.81 (14)	C8—C7—C12—C11	0.0 (3)
O1—P1—N1—C13	−179.07 (15)	O3—C7—C12—C11	178.59 (17)
O2—P1—N1—C13	51.11 (18)	C10—C11—C12—C7	−0.3 (3)
O3—P1—N1—C13	−51.04 (18)	P1—N1—C13—C18	−0.3 (3)
P1—O2—C1—C6	5.8 (3)	P1—N1—C13—C14	179.39 (14)
P1—O2—C1—C2	−175.33 (13)	C18—C13—C14—C15	0.1 (3)
C6—C1—C2—C3	−0.8 (3)	N1—C13—C14—C15	−179.59 (17)
O2—C1—C2—C3	−179.63 (17)	C13—C14—C15—C16	−0.4 (3)
C1—C2—C3—C4	0.6 (3)	C14—C15—C16—C17	0.1 (3)
C2—C3—C4—C5	−0.1 (3)	C14—C15—C16—C19	−179.19 (19)
C3—C4—C5—C6	−0.2 (3)	C15—C16—C17—C18	0.4 (3)
C2—C1—C6—C5	0.4 (3)	C19—C16—C17—C18	179.8 (2)
O2—C1—C6—C5	179.22 (18)	C16—C17—C18—C13	−0.7 (3)
C4—C5—C6—C1	0.0 (3)	C14—C13—C18—C17	0.4 (3)
P1—O3—C7—C8	−86.63 (19)	N1—C13—C18—C17	−179.86 (19)
P1—O3—C7—C12	94.78 (19)		

### Hydrogen-bond geometry (Å, °)

**Table:**

*D*—H···*A*	*D*—H	H···*A*	*D*···*A*	*D*—H···*A*
N1—H1···O1^i^	0.79	2.07	2.840 (3)	167

Symmetry codes: (i) −*x*+1, −*y*+1, −*z*+1.
